# Near Infrared Light Treatment Reduces Synaptic Levels of Toxic Tau Oligomers in Two Transgenic Mouse Models of Human Tauopathies

**DOI:** 10.1007/s12035-018-1248-9

**Published:** 2018-08-17

**Authors:** Michele M. Comerota, Batbayar Tumurbaatar, Balaji Krishnan, Rakez Kayed, Giulio Taglialatela

**Affiliations:** University of Texas Medical Branch, 301 University Blvd., Galveston, TX 77555-1045 USA

**Keywords:** Near infrared light, Tau oligomers, hTau mouse, 3xTgAD mouse, Autophagy

## Abstract

Tau oligomers are emerging as a key contributor to the synaptic dysfunction that drives cognitive decline associated with the clinical manifestation and progression of Alzheimer’s disease (AD). Accordingly, there is ample consensus that interventions that target tau oligomers may slow or halt the progression of AD. With this ultimate goal in mind, in the present study, we investigated tau oligomer accumulation and its synaptic and behavioral consequences after an in vivo treatment with near infrared (NIR) light (600–1000 nm) in two transgenic mouse models, overexpressing human tau either alone (hTau mice) or in combination with amyloid beta (3xTgAD mice). We found that a 4-week exposure to NIR light (90 s/day/5 days a week) significantly reduced levels of endogenous total and oligomeric tau in both synaptosomes and total protein extracts from the hippocampus and cortex of hTau mice and improved deteriorating memory function. Similar results were observed in the 3xTgAD mice, which further displayed reduced synaptic Aβ after NIR light treatment. On the other hand, ex vivo binding of tau oligomers in isolated synaptosomes as well as tau oligomer-induced depression of long-term potentiation (LTP) in hippocampal slices from NIR light-treated wt mice were unaffected. Finally, levels of proteins critically involved in two mechanisms associated with clearance of misfolded tau, inducible HSP70 and autophagy, were upregulated in NIR light treated mice. Collectively, these results show that NIR light decreases levels of endogenous toxic tau oligomers and alleviate associated memory deficits, thus furthering the development of NIR light as a possible therapeutic for AD.

## Introduction

Alzheimer’s disease (AD) is the most frequent age-related dementia, for which there is currently no resolving cure. The multifactorial nature of AD has contributed to the ongoing challenge of developing effective disease-modifying therapeutics. The accumulation of plaques and neurofibrillary tangles (NFTs) consisting of amyloid beta (Aβ) and hyperphosphorylated tau protein, respectively, are two quintessential hallmarks of AD. However, many factors such as mitochondrial dysfunction, neuroinflammation, impaired clearance of dysfunctional proteins, and synaptic retraction contribute to the disease progression [1–4]. Among those factors, synaptic dysfunction is believed to underlie the onset and progression of the cognitive impairment that characterizes the symptomatic phase of AD [5]. Emerging evidence suggests that the small soluble oligomeric aggregate forms of tau contribute to the dysfunction of the synapses by acting both intracellularly and extracellularly [6, 7]. Indeed, while accumulations of NFTs have been found to strongly correlate with the decline of cognitive function [8, 9], compelling evidence points at tau oligomers as the most toxic form of tau aggregates [10, 11]. After the hyperphosphorylation of the tau protein, aggregates mislocalize from the axonal to the somatodendritic region [12]. This increased concentration of synaptic tau oligomers interferes with the process of synaptic transmission [6]. In addition, tau oligomers released to the extracellular space have also been suggested to act on the postsynaptic regions resulting in increased intracellular calcium [13] and impaired long-term potentiation (LTP) [7]. Further, tau oligomers act in a prion-like manner, seeding the misfolding and aggregation of cellular monomeric tau and resulting in the spreading of the disease from cell to cell [14]. Together, this evidence suggests that the targeting of tau oligomer accumulations, specifically at the synapses, will mitigate the synaptic dysfunction and the progression of clinical manifestation of AD.

With this ultimate goal in mind, we investigated the administration of non-invasive, transcranial near infrared (NIR; 600–1000 nm) light as a potential treatment for AD in relevant transgenic mouse models. Previous studies have found a reduction of hyperphosphorylated tau and NFTs in NIR light-treated K369I mice [15], a model of frontotemporal dementia [16]. In addition, we and others have previously shown a direct effect of NIR light in reducing pathological Aβ accumulations in transgenic mouse models of APP overexpression and further described increased synaptic resilience to the binding and dysfunctional impact Aβ oligomers in NIR light-treated mice [15, 17–19]. Despite this suggestive initial evidence, however, the effect of NIR light on levels of total and synaptic tau oligomers as well as synaptic tau oligomer-induced functional changes remains unknown. In the present study, we aimed to determine whether NIR light could promote the reduction of synaptic accumulation of tau in two tg mouse models of human tauopathies (hTau and 3xTgAD) in vivo and thus offer neuroprotection against the dysfunctional tau oligomer synaptic binding. We further investigated autophagy-related proteins and the chaperone inducible heat shock protein 70 (HSP70) as possible mechanisms mediating clearance of tau in response to NIR light treatment. We show that, when applied to either of these transgenic mouse models of human tauopathies, NIR light effectively clears toxic tau oligomers, both from the CNS parenchyma and the synapse, and restores memory functions in these impaired mice. Overall, these novel results illustrate a direct effect of NIR light in clearing the CNS from toxic tau oligomers, thus supporting the notion that NIR light should be further explored as a non-invasive therapeutic strategy in AD and related tauopathies.

## Results

### Reduced Tau Pathology in Cortical and Hippocampal Total Protein Extracts and Synaptosomal Fractions of NIR Light-Treated 13-Month-Old hTau Mice

**Fig. 1:**
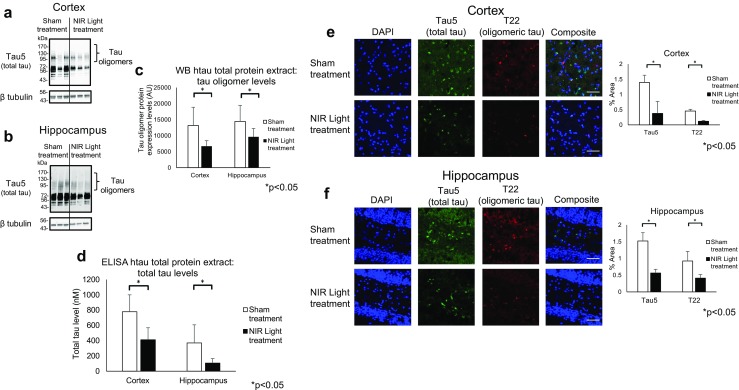
Reduced total and oligomeric tau in cortical and hippocampal total protein extract of 13-month hTau mice. Western blot, ELISA, and fluorescence immunohistochemistry were used to measure total tau and oligomeric tau levels in the total protein extracts of hTau mice treated with NIR light (filled columns) and hTau mice receiving the sham no light treatment (open columns). Representative Western blots of total tau levels, using the Tau5 antibody, in the total protein extract of the **a** cortex and the **b** hippocampus of NIR light and sham treated hTau mice. **c** Oligomeric tau levels were measured by calculating the densitometry of the Western blot bands greater than 110 kDa. **d** The total tau levels in the total protein extracts of the cortex and hippocampus, using Tau5 antibody, was also quantitatively assessed by ELISA. Immunofluorescence was used to further measure the total and oligomeric tau levels. Total tau levels were measured using the total tau specific antibody, Tau5, and oligomeric tau levels were measured using the oligomer tau specific antibody, T22, in the **e** cortex and **f** hippocampus of the treated hTau mice. Student’s two tailed *t* test was used to determine statistical significance. (*n* =6 per group). Error bars represent SD. *p<0.05

**Fig. 2:**
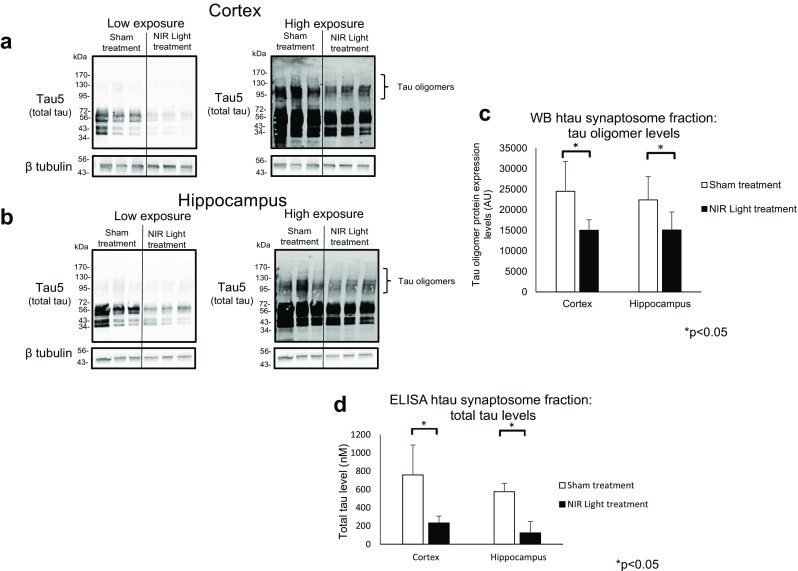
Reduced total and oligomeric tau at cortical and hippocampal synapses of 13-month hTau mice. Total and oligomeric tau was measured by Western blot and ELISA analysis in the cortical and hippocampal synaptosome fractions of hTau mice treated with NIR light. Representative Western blots of the total tau levels, using the Tau5 antibody, in the **a** the cortical synaptosomes and **b** the hippocampal synaptosomes of NIR light- and sham-treated mice. The membranes were analyzed using different exposure rates; low exposure, to properly visualize the monomeric tau band, and high exposure, to visualize the oligomeric band of tau. **c** The oligomeric tau band was measured in the high exposure  rate of the membrane. **d** The total tau levels in the synaptosomes fractions of the cortex and hippocampus was also analyzed by ELISA analysis, using the total tau antibody, Tau5. Student’s two-tailed *t* test was used to determine statistical significance. (*n*=6; per group). Error bars represent SD. *p 0.05

We aimed to determine if NIR light reduces the in vivo synaptic accumulation of endogenous tau oligomers by utilizing the transgenic human tau mouse model, hTau. This well-characterized mouse model develops tau aggregates, including abundant tau oligomers, around 9–10 months of age [20, 21]. Therefore, to ensure adequate tau accumulation prior to our intervention, we began NIR light treatment at 12 months of age (*n*=7; per group). The total protein extracts and the isolated synaptosomal fractions of the cortex and hippocampus regions were analyzed by Western blot (Fig. 1a–c, Fig. 2a–c; respectively), ELISA (Fig. 1d, Fig. 2d; respectively), and fluorescence immunohistochemistry (IHC) (Fig. 1e, f) to qualitatively and quantitatively determine levels of tau oligomers and total tau (including both monomeric and oligomeric species). Tau oligomers were quantitatively measured by densitometry analysis of Western blot bands of 110 kDa and higher (Fig. 1a–c) as detected by the total tau antibody, tau5. In the cortical and hippocampal total protein extracts, we found a decrease in tau oligomers in NIR light-treated hTau mice compared to sham-treated control mice (cortex *p*=0.016, hippocampus *p*=0.049). We further analyzed the total tau levels in the total protein extracts utilizing a tau5 ELISA analysis. As shown in Fig. 1d, there was a statistically significant decrease in the total tau in both the cortex and hippocampus (cortex *p*=0.049, hippocampus *p*=0.049). Finally, these results were confirmed by fluorescence IHC analysis of the hippocampus and cortex regions using antibodies specific for tau oligomers, T22 and total tau, tau5. The immunofluorescence further verified a reduction of both total tau (cortex *p*=0.002, hippocampus *p*=0.032) and tau oligomers (cortex *p*=0.003, hippocampus *p*=0.045) in both regions (Fig. 1e, f). We then analyzed the levels of total and oligomeric tau in the synaptosomal fractions by Western blot and ELISA analysis to determine whether the reduction of tau observed in the total protein extracts was paralleled by a similar reduction at the synaptic compartment. Due to technical challenges in imaging simultaneously all forms of tau in the synaptosomal regions of the hTau mice on the same Western blot, two different exposures were taken to properly visualize monomeric tau (low exposure) and oligomeric tau (high exposure) (Fig. 2a, b). Tau oligomer levels were reduced in synaptosomal fractions of both cortex and hippocampus of NIR light-treated hTau mice compared to the sham-treated mice (cortex *p*=0.049, hippocampus *p*=0.049) (Fig. 2c). Further, levels of total tau, as measured by tau5 ELISA, were also reduced in the cortical and hippocampal synapses of these mice (Fig. 2d) (cortex *p* =0.024, hippocampus *p* = 0.003). Collectively, these results suggest a systemic, as well as, synaptic reduction of total and oligomeric tau in hTau transgenic mice treated with NIR light.

### Rescue of Impaired Long-Term Memory in Aged NIR Light-Treated hTau Mice

**Fig. 3:**
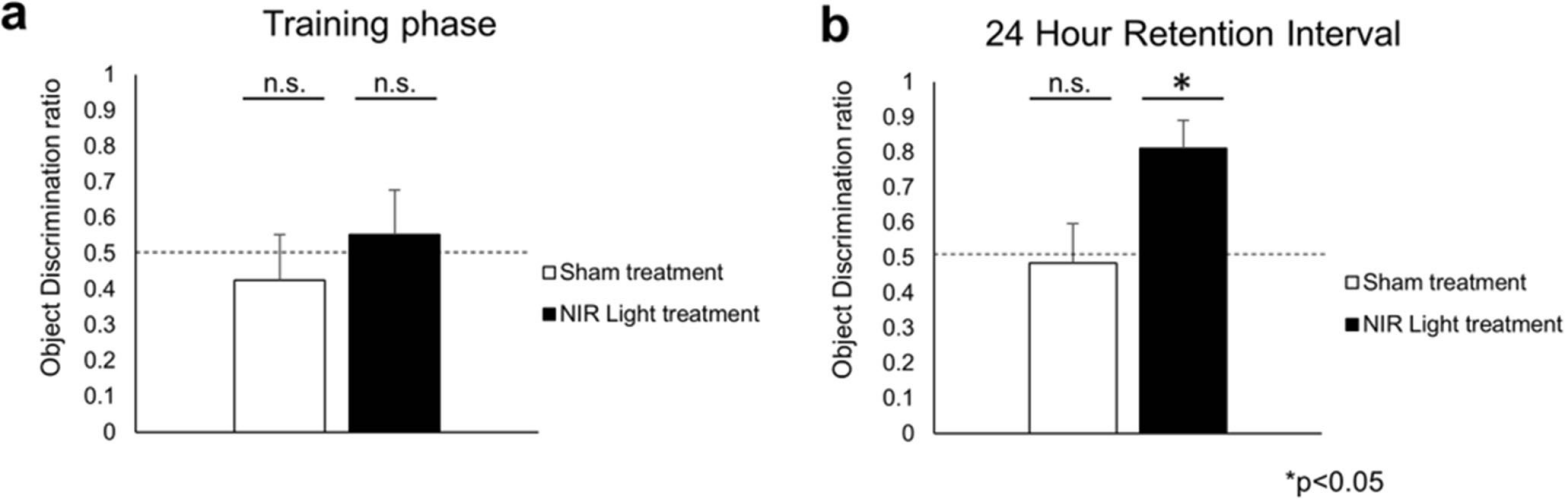
Improved memory in NIR light treat hTau mice. The novel object recognition (NOR) test was used to determine the long-term memory of hTau mice that received NIR light (filled columns) or sham (open  columns) treatments (*n* = 6; per group). **a** The object discrimination ratio during the training phase (in which two identical objects are placed in the open field) was calculated by the time spent with the object placed in the quadrant of the future novel object divided by the total time spent exploring the two identical objects. **b** The object discrimination ratio during the 24-h test was calculated by the time spent with the novel object divided by the total time exploring the novel and familiar object. The object discrimination ratios of each group were analyzed by the one-sample t test to determine the statistical variation from chance (0.50). n.s. represents not statistically significant significance *p < 0.05

Because of the reduction in tau oligomers observed after NIR light treatment in hTau mice, we next aimed to determine if such reduction would translate into a functional benefit in these mice. To determine if the cognitive function improved in hTau mice treated with NIR light, we performed the novel object recognition (NOR) paradigm immediately following the last NIR light treatment. During the training phase, the mice were allowed to freely explore for 10 min two identical objects placed in the testing arena. We found no difference between hTau mice treated or not with NIR light in the time spent exploring the objects during the training phase, which was equally split between the two identical objects (Fig. 3a). To determine long-term memory, 24 h after the training phase was completed, one of the two objects was replaced by a novel object and the mice were again allowed to freely explore the objects. Based on the propensity of mice to spend more time exploring an object they have not explored before, an extended amount of time exploring the novel object reflects memory of the familiar object. The object discrimination ratio (ODR) was calculated to determine the percentage of time spent with the novel object (Fig. 3). We found the animals treated with NIR light had an ODR of about 0.8 (one sample *t* test, *p*=0.0001) of the novel object indicating an increased exploration of the novel object compared to the familiar object. On the other hand, the hTau mice receiving no treatment had a ODR of about 0.48 (one sample *t* test, *p*=0.765), indicating no difference in the exploration time between the novel and familiar object and thus reflecting memory impairments as previously described in aged hTau mice [22]. This suggests a recovery of impaired memory function hTau mice that received the NIR light treatment.

### Reduced Tau and Aβ Pathology in the Cortex and Hippocampus of NIR Light-Treated 13-Month Old 3xTgAD Mice

**Fig. 4:**
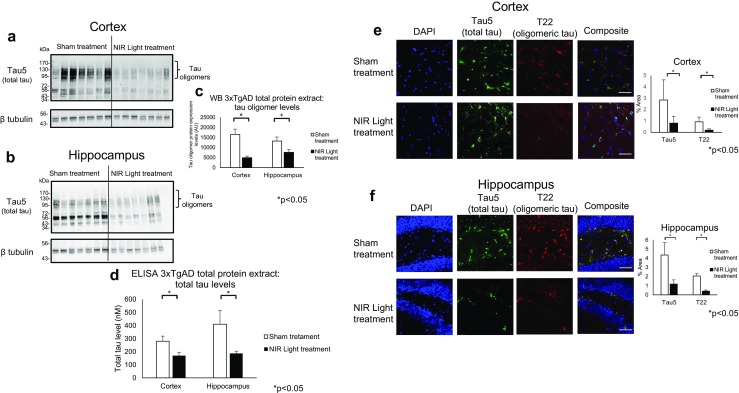
Reduced total and oligomeric tau in cortical and hippocampal total protein extracts of 13-month 3xTgAD mice. The total protein extracts from the cortex and hippocampus of 3xTgAD mice receiving NIR light treatment (filled columns) or sham treatment (open columns) were analyzed by Western blot, ELISA, and immunofluorescence analysis. Oligomeric tau levels were determined by measuring the bands displayed at 110 kDa and above on Western blots of total tau levels, using the Tau5 antibody, in the total protein extract of the **a**, **c** cortex and the **b**, **c** hippocampus of NIR light- and sham-treated 3xTgAD mice. **d** ELISA analysis using the total tau antibody, Tau5, was used to measure the levels of all forms of tau in the cortex and hippocampus. Further, immunofluorescence with the Tau5 and the tau oligomer specific antibody, T22 was used to determine the total and oligomeric tau levels in the **e** cortex and **f** hippocampus. Student’s two-tailed *t* test was used to determine statistical significance. (*n*=7; per group). Error bars represent SD. *p<0.05

**Fig. 5:**
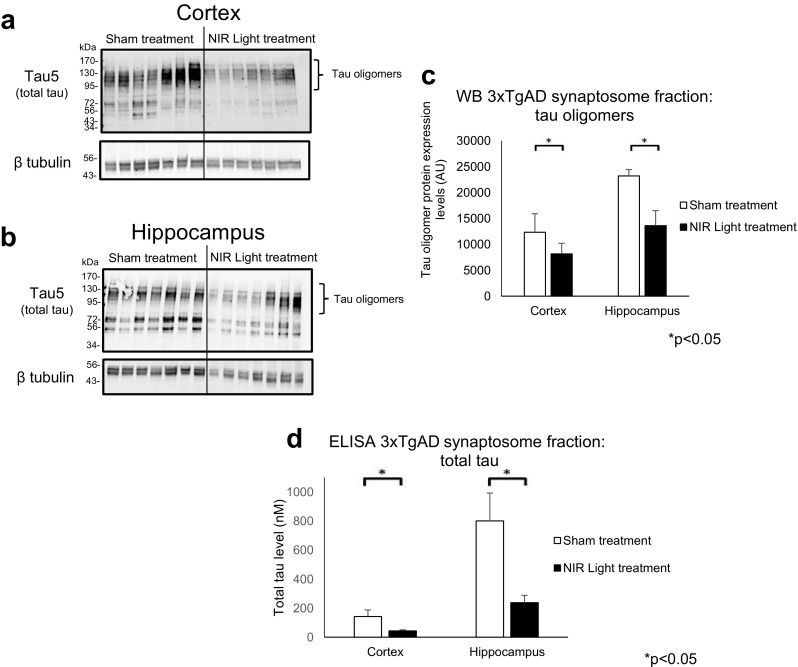
Reduced total and oligomeric tau at cortical and hippocampal synapses of 13-month 3xTgAD mice. The total and oligomeric tau levels in the synaptosomal region of 3xTgAD mice receiving NIR light (filled columns) and sham (open columns) treatments were measured by Western blot and ELISA analysis. Representative Western blots of total tau levels, Tau5 antibody, in the synaptosomes of the **a** cortex and the **b** hippocampus of NIR light- and sham-treated 3xTgAD mice. **c** Western blot bands greater than 110 kDa were measured to determine the levels of the oligomeric form of tau. **d** The total tau levels in the total protein extracts of the cortex and hippocampus was also analyzed through tau5 ELISA analysis. Student’s two-tailed *t* test was used to determine statistical significance. (*n*=7; per group). Error bars represent SD. *p<0.05

**Fig. 6:**
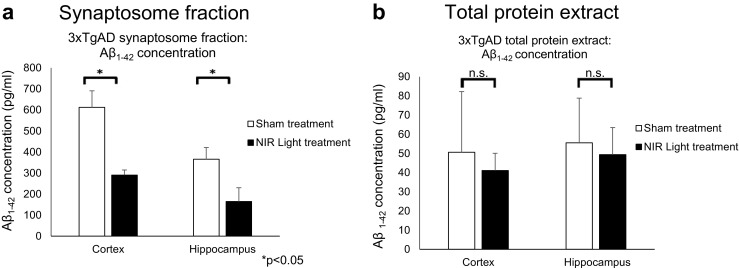
Amyloid beta levels are decreased in the synapses of 3xTgAD mice treated with NIR light. Aβ levels in **a** synaptosome fraction and **b** the total protein extract from the cortex and of NIR light (filled columns) and sham (open columns) treated 3xTgAD mice (*n*=7; per group) were measured using ELISA analysis. Student’s two-tailed *t* test was used to determine statistical significance. Error bars represent standard deviation. *p<0.05; n.s. represents not statistically significant

In order to determine if NIR light treatment results in a reduction of both Aβ and tau when they are co-expressed in a combined endogenous system, we investigated changes in both Aβ and tau oligomers at the synapses and in total protein extracts of 3xTgAD mice. The 3xTgAD mice model exhibit overexpression of three AD-relevant human genes; human APP bearing the Swiss mutation, human tau with a P301L mutation, and presenilin-1 with the M146V mutation, resulting in extensive aggregated Aβ and tau pathology around 12 months of age [23]. We therefore began the 4 week-long NIR light treatment at 12 months of age to ensure that accumulations of both toxic proteins had already occurred when treatment was applied (*n*=7; per group). We first employed Western blot, ELISA, and fluorescence IHC analyses to determine levels of total and oligomeric tau in total protein extracts from the cortical and hippocampal regions of 3xTgAD mice receiving NIR light treatment (Fig. 4). Densitometry analysis of the bands >110 kDa (tau oligomers) detected in Western blots by the tau5 antibody (Fig. 4a, b) revealed a statistically significant decrease of tau oligomers in both brain regions of the NIR light-treated mice as compared to the sham treated animals (cortex *p*=0.001, hippocampus *p*=0.047) (Fig. 4c). We further measured total tau levels using the tau5 ELISA and found a similar decrease in both brain regions of the NIR light-treated mice (cortex *p*=0.038, hippocampus *p*=0.049) (Fig. 4d). We further used fluorescence IHC to confirm the reduction of tau oligomers, using the tau oligomer specific antibody T22, and total tau, using the total tau antibody tau5 (Fig. 4e, f). We found a decrease of both oligomeric and total tau in the hippocampus (*p*=0.001, *p*=0.045) and cortex (*p*=0.007, *p*=0.045) of NIR light-treated mice as compared to the sham treated. We next measured total and oligomeric tau levels in the synaptosomal fractions of NIR light-treated 3xTgAD mice by Western blot and ELISA. Western blot analysis revealed a decrease in the levels of oligomeric tau in the cortex (Fig. 5a, c) and hippocampus regions (Fig. 5b, c) (cortex *p*=0.009, hippocampus *p*=0.038). In addition, a significant decrease of total tau levels in the synaptosomal fractions was observed in the cortex and the hippocampus (cortex *p*=0.039, hippocampus *p*=0.015) of NIR light-treated mice, as measured by tau5 ELISA (Fig. 5d). Finally, we employed a Aβ_1–42_-specific ELISA to measure the levels of Aβ in the synaptosomal fractions and total protein extracts from the cortex and hippocampus. As shown in Fig. 6a, Aβ levels were reduced in the synaptosomal fractions from the cortex and hippocampus regions of NIR light-treated mice as compared to sham animals (cortex *p*=0.002, hippocampus *p*=0.040). However, the levels of Aβ in the total protein extracts were unchanged in the NIR light-treated group compared to the sham-treated group (cortex *p*=0.495, hippocampus *p*=0.145) (Fig. 6b). Collectively, these results show a similar decrease in the tau and Aβ levels induced by the NIR light treatment in the 3xTgAD mice.

### NIR Light Treatment Does Not Affect Ex Vivo Synaptic Binding of Tau Oligomers

**Fig. 7:**
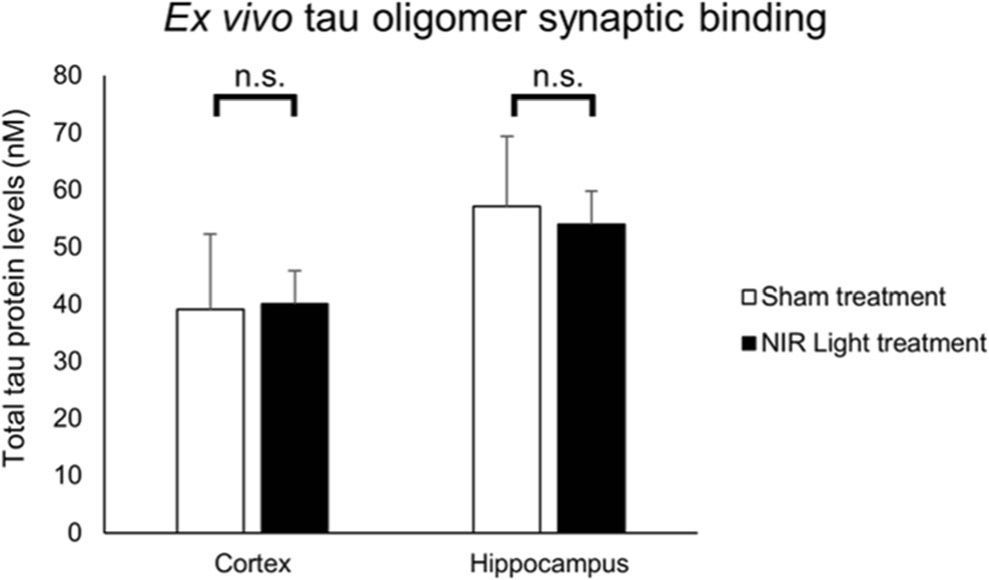
Synaptic binding of tau oligomers is not altered in NIR light-treated WT mice. ELISA analysis was conducted on the ex vivo challenge of synaptosomes isolated from the cortex and hippocampus of NIR light- and sham-treated wild-type mice with 50 nM of tau oligomers (*n*=8; per group). Statistical significance was determined by Student’s two-tailed *t* test analysis. Error bars represent standard deviation. n.s. represents not statistically significant

Recent studies have indicated that extracellular tau oligomers play a key role in the induction of synaptic dysfunction [7]. In the current study, we aimed to determine if NIR light induces a reduction of synaptic vulnerability to tau oligomers thus preserving cognitive function. We performed an ex vivo tau binding study in which synaptosomes isolated from wild-type mice that received either NIR light or sham treatment (*n*=7, per group) were exposed to 50 nM of tau oligomers for 1 h, as described in the “Methods” section. After washing off unbound oligomers, the remaining levels of tau protein bound to synaptosomes were measured by ELISA analysis. We found that both cortical and hippocampal synaptosomes from NIR light-treated mice bound similar levels of exogenously added tau oligomers as compared to the sham-treated mice (cortex *p*=0.514, hippocampus *p*=0.867) (Fig. 7). This suggests that NIR light treatment does not affect the susceptibility of the synapses to the association with tau oligomers.

### Suppression of Hippocampal Long-Term Potentiation by Tau Oligomers Is Not Reversed by NIR Light Treatment

**Fig. 8:**
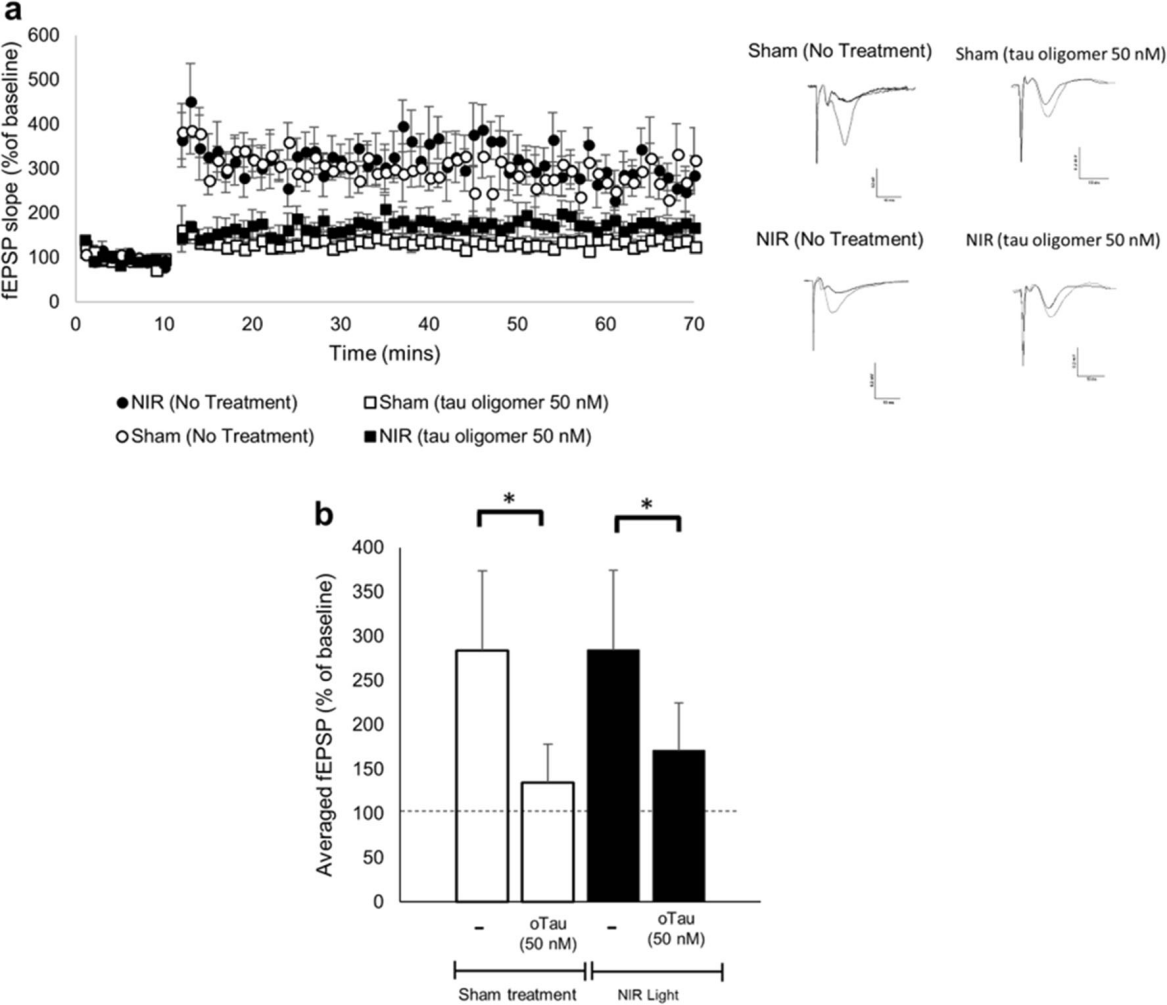
Tau oligomer induced impairment of LTP is not altered in NIR light-treated mice. The long-term potentiation (LTP) was measured by Schaffer collateral field recordings to determine the impact of NIR light treatment on tau oligomer induced impairments in wild-type mice. **a** The fEPSP amplitude was calculated for the four groups; NIR light treated with and without tau oligomers and sham treated with and without tau oligomers. (*n*=5; per group, two slices per condition). **b** For each group, the calculated fEPSP of the final 10 min of recording was averaged. One-way ANOVA with Dunn’s post hoc analysis was used to determine statistical significance. Error bars represent ±standard error of mean. *p<0.05

Although we found that NIR light treatment induced no changes in synaptic susceptibility to tau oligomer binding, we aimed to determine if NIR light provides protection against tau oligomer-induced synaptic dysfunction. Hippocampal slices prepared from wild-type mice that were exposed to NIR light or sham treatment were added with 50 nM of tau oligomers (*n*=5 per treatment group, two slices per condition from each animal), a concentration known to impair long-term potentiation (LTP) [7] and determine the extent of LTP expression after high-frequency stimulation in the Schaffer collateral pathway (Fig. 8). The last 10 min of LTP were averaged for each treatment group (Fig. 8b) and evaluated for statistical differences among groups. Consistent with the literature, we found a statistically significant reduction in the magnitude of LTP in the slices from sham-treated mice exposed to 50 nM of tau oligomers (*p*=0.001). We also found a reduction in the magnitude of LTP in slices prepared from the NIR light-treated group exposed to tau oligomers compared to similar slices that however were not exposed to tau oligomers (*p*=0.01). We further found that there was no difference between the NIR light-treated and untreated groups in the extent of the reduction of LTP induced by tau oligomer exposure (*p*>0.05). Lastly, the basal synaptic strength, as measure by input-output curves, was not changed in any of the treatment group (data not shown). Collectively, these results suggest that NIR light treatment does not affect vulnerability of synapses to tau oligomer-induced LTP impairments.

### Increased Inducible HSP70 at the Synapses of NIR Light-Treated 3xTgAD, hTau, and Wild-Type Mice

**Fig. 9:**
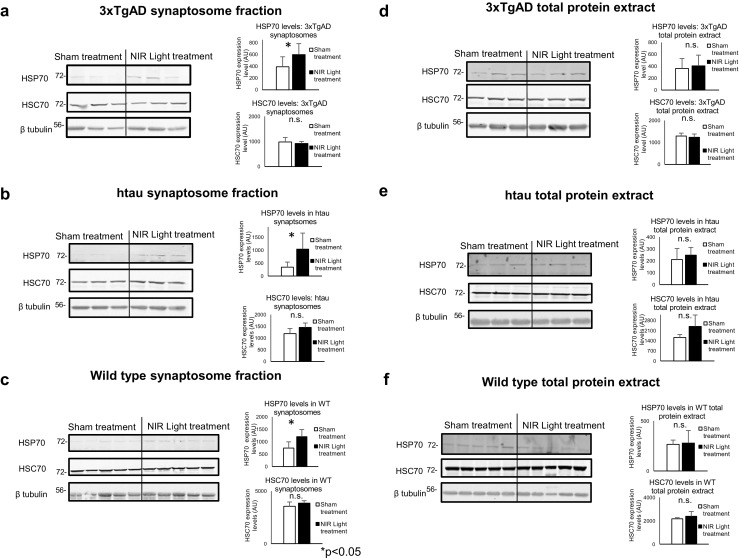
HSP70 is increased in the synaptosomal fractions but not in the total protein extracts of wt, 3xTgAD and hTau mice. Representative Western blot analysis of inducible HSP70 (iHSP70) and HSC70 protein levels in synaptosome fractions and total protein extracts from the hippocampus of **a** 3xTgAD (*n*=7; per group), **b** hTau (*n*=6; per group), and **c** wild-type (*n*=7; per group) mice treated (filled columns) or not (open columns) with NIR light. Error bars represent standard deviation. *p<0.05 (Student’s two-tailed *t* test)

The results of our experiments suggested that NIR light effectively induces a significant reduction of the levels of both total and oligomeric tau, suggesting increased clearance. To investigate a potential mechanism for this putative clearance, we measured heat shock protein 70 (HSP70) levels in wild-type, 3xTgAD, and hTau mice after NIR light treatment. We elected to investigate HSP70 because previous studies have described the intimate relationship between upregulation of inducible HSP70 and the reduction of tau in animal models [24]. We utilized Western blot analysis to measure the levels of inducible HSP70 as well as its constitutive isoform, heat shock cognate 70 (HSC70), in the synaptosomal fractions and total protein extracts from brains of mice exposed to a 4-week treatment with NIR light. We found that in all three animal models, there was an increase of HSP70 levels in the synaptosomal fractions (Fig. 9a–c) ((a) *p*=0.049, (b) *p*=0.034, (c) *p*=0.029) but no change in the total protein extract (Fig. 9d–f) ((d) *p*=0.651, (e) *p*=0.127, (f) *p*=0.844). On the other hand, there was no change in the levels of the constitutively active HSC70 in either the total protein or the synaptosome fractions of the NIR light treated mice ((a) *p*=0.664, (b) *p*=0.143, (c) *p*=0.268, (d) *p*=0.401, (e) *p*=0.084, (f) *p*=0.275). These results indicate a selective increase of the inducible HSP70 at the synapse in NIR light-treated mice.

### Increased Levels of Autophagy Markers in NIR Light-Treated 3xTgAD

**Fig. 10:**
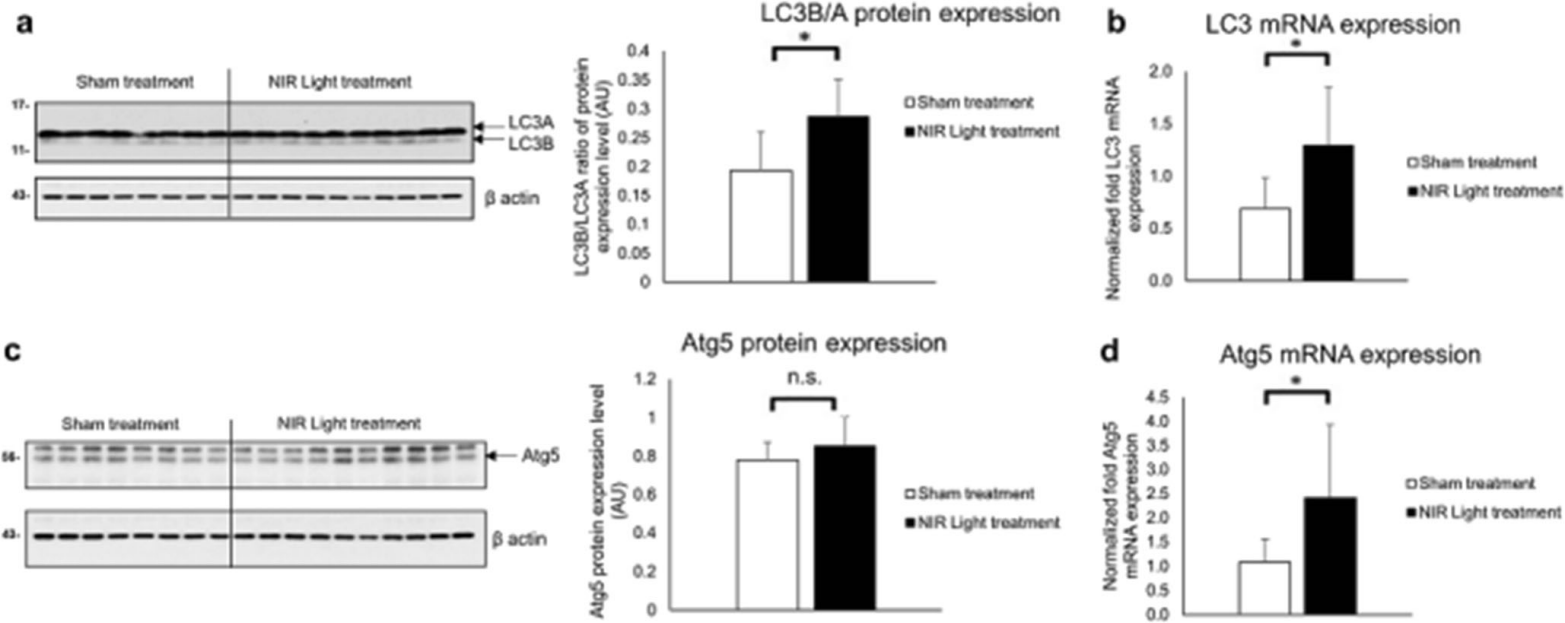
Upregulation of autophagy markers in total protein extracts after NIR light treatment. Protein and mRNA levels of autophagy markers in the total protein extracts of NIR light (filled columns) (*n*=9) and sham treated (open columns) (*n*=7) 3xTgAD mice as determined by Western blot analysis and RT-PCR. **a** Representative Western blot detecting LC3A and LC3B proteins and quantitative densitometry analysis of the LC3B/A ration. **b** LC3 and **c** Atg5 mRNA expression levels as measured by RT-PCR. Error bars represent standard deviation. *p<0.05 (Student’s two-tailed *t* test)

To further gain an understanding of the contributing mechanisms to the NIR light-induced reduction of tau in the transgenic mice models, we measured the protein and mRNA levels of LC3A and B, and Atg5, proteins that are known to be key in the initiation of autophagy [25]. We found that the levels of LC3B were increased (*p*=0.001) in the NIR light-treated mice (*n*=8) compared to sham-treated mice (*n*=10), whereas the levels of LC3A remained unchanged (0.992) between the treatment groups (Fig. 10a). The resulting increased ratio between LC3B and LC3A (*p*=0.008) in the NIR light-treated group suggests an increased promotion of autophagosome formation [26]. In addition, the mRNA levels of LC3 were also increased (Fig. 10b). The protein expression levels of Atg5 showed a trend of increase in NIR light-treated mice that however did not reach statistically significance (Fig. 10c). On the other hand, Atg5 mRNA levels were significantly increased (*p*=0.044) in the NIR light-treated 3xTgAD (Fig. 10d). These results suggest NIR light-treated 3xTgAD have increased expression of proteins that contribute to the induction of autophagy.

## Discussion

Impairment of synaptic function is a key event that initiates the progressive cognitive decline that is associated with clinically manifest AD [5, 27]. The oligomeric form of the microtubule-associated protein tau is emerging as a key contributor to the disruption of synaptic function in AD [28–30]. The main goal of our study was to determine if NIR light induces neuroprotection against the synaptic association and accumulation of the toxic tau oligomers, as well as the subsequent oligomer-driven dysfunction. We first aimed to determine if the administration of NIR light treatments in two mouse models of tauopathies, hTau (accumulation of tau) and 3xTgAD (accumulation of Aβ and tau), would reduce the synaptic accumulation of tau oligomers. Because of the complex relationship between Aβ and tau with extensive evidence that these proteins can influence the pathology of each other, it is critical to examine the proteins in a combined mouse model, as well as, in a simpler mouse model where tau is singly overexpressed. Previous research has found a general reduction of hyperphosphorylated tau in the Parkinsonian mouse model K369I upon exposure to NIR light [15]. However, whether this NIR light-induced reduction of tau would hold true for tau oligomers and their synaptic accumulation (a major determinant of tau neurotoxicity) remained unexplored. In physiological conditions, tau is localized to the axonal region of neurons with low levels in the synaptic regions contributing to the stability of protein scaffolding. However, in AD, tau relocalizes to the somatodendritic region. The elevated levels of tau oligomers at the synapses are believed to contribute to the interference of synaptic function [12, 31, 32]. The hTau mice, a mouse that expresses a human MAPT transgene and knockout of mouse MAPT, served as a model that exclusively expresses the accumulation of tau protein [20]. Our results showed not only a reduction of tau oligomer levels at the cortex and hippocampus synapses of NIR light-treated animals but also in the total protein extracts. We further found that total tau levels, which include monomers, as well as all aggregated tau species, were also decreased in both the synapses and total protein extracts. While previous studies have suggested the knockout of tau in mice can result in degeneration of the axons [33], the decreased observed in this study does not eliminate all tau suggesting that an impairment to the structural system of the neurons will not be greatly impacted. In addition, this decrease seems to include monomeric tau, which can act as seeds for the formation of oligomeric tau suggesting this decrease in total tau may be beneficial [34]. Thus, unlike our previous results in which we found that NIR light induces a selective reduction of Aβ oligomers at the synapses in the human amyloid precursor protein (APP) overexpressing mouse model, Tg2576 [19], the tau reduction in the hippocampus and cortex of NIR light-treated hTau mice that we observed in the present study is not limited to the synaptic region, but rather occurs throughout the brain parenchyma. Nonetheless, this molecular phenomenon of overall reduced tau oligomers translates into a functional benefit as illustrated by the observed memory improvement in the novel object recognition (NOR) test of hTau mice that received the NIR light treatment. This mouse model is known to have memory deficits as measured by NOR at 10 months of age [22]. The NIR light-treated hTau displayed an improved performance compared to the sham-treated hTau mice, as measured by the increased time spent with the novel object. This test indicates that the NIR light treatment ameliorated the cognitive impairment typically displayed by hTau mice at this age. Collectively, our data show that NIR light treatment reduced the tau pathology and the corresponding memory deficits in hTau mice.

The second mouse model we utilized, 3xTgAD, exhibits overexpression of three genes associated with familial AD; presenilin 1, human tau with the P301L mutation, and human APP with the Swedish mutation. These mice display deposits of both aggregated Aβ and tau by 12 months of age, serving as a model for the simultaneous presence of these toxic proteins as observed in AD [23]. We found that both total and oligomeric tau was reduced in the synaptic compartment as well as in the total protein extract in 3xTgAD mice, whereas the Aβ was reduced exclusively at the synapses. This is similar to what we observed here in the hTau mice, as well as we previously reported in the Tg2576 mice [19]. Our results thus suggest that the NIR light-induced mitigation of Aβ and tau pathology is sufficient and equally effective when the two amyloid proteins coexist in a system, supporting NIR light as a promising treatment for AD.

We next aimed to determine if NIR light induces neuroprotective synaptic resistance to the association of extracellular tau oligomers and tau oligomer-induced synaptic dysfunction. We utilized an ex vivo binding approach to determine if synaptosomes isolated from NIR light-treated wt mice displayed an altered affinity to exogenously applied tau oligomers. The results showed an equivalent tau association to the synapses isolated from the NIR light-treated group compared to the sham-treated mice.

We previously reported that NIR light treatment in wt mice reduced synaptic vulnerability to Aβ oligomers, both in terms of binding and Aβ oligomer-induced suppression of hippocampal LTP expression [19]. Therefore, in the present studies, we further measured LTP in the Schaffer collateral pathway of the hippocampus after the application of tau oligomers to brain slices prepared from these NIR light-treated wt mice. Application of tau oligomers to brain slices has previously been shown to induce a significant impairment of hippocampal LTP induction/expression [7]. As in the case of the tau oligomer synaptic binding experiments described earlier, incubation of brain slices with tau oligomers evoked a similar impairment in LTP expression in the NIR light-treated group and the sham. Contrary to what we previously observed for Aβ oligomers, these results suggest that NIR light does not induce mechanisms that reduce the synaptic vulnerability to and functional impact of tau oligomers. This could indicate that tau and Aβ oligomers act on the synapses in differing ways. While Aβ oligomers are known to have multiple synaptic binding partners such as mGluR5 and α7-nicotinic acetylcholine receptors among others [35, 36], the mechanisms by which extracellular tau oligomers associate with the synapses remain elusive. Previous studies have implied that the similar structures of Aβ and tau oligomers contribute to similar mechanisms of toxicity [37]. However, our combined studies suggest that NIR light alters mechanisms that exclusively contribute to Aβ oligomer synaptic association.

While the primary mechanisms of action of the NIR light in stimulating bioenergy output and efficiency of mitochondria has been well established, the secondary mechanisms that lead to neuroprotection remains poorly understood [38]. The systemic reduction of both total and oligomeric tau in NIR light-treated hTau mice that we report here suggests that NIR light initiates mechanisms that contribute to the regulation and the clearance of tau. In the current study, we investigated the chaperone HSP70 and proteins involved the induction of autophagy as two known pathways that have been reported to promote the degradation of dysfunctional tau [2, 39]. The HSP70 family of proteins are chaperones that are involved in the refolding and shuttling of dysfunctional proteins to degradation pathways [40]. There is extensive literature describing the intimate relationship between inducible HSP70 and the clearance of the toxic tau proteins. Particularly, evidence demonstrates the reduction of aggregated tau after stimulating the activity of or overexpressing endogenous inducible HSP70 or following administration of exogenous inducible HSP70 [39, 41, 42]. On the other hand, an opposite correlation exists between tau and heat shock cognate 70 (HSC70), a constitutively expressed member of the HSP70 family, whereby HSC70 overexpression slows the clearance of misfolded tau deposition [24]. The observed exclusive synaptic increase of inducible HSP70 and unchanged levels of HSC70 after NIR light treatment that we observed in all animal models surveyed here suggests specificity of the phenomenon. Such increase could indicate a specific induction of HSP70 in neurons or possibly reflect the relocalization of inducible HSP70 to the synapses as a means of protecting the compartment from future insults. We further investigated the autophagy pathway, one of the pathways that HSP70 has been shown to shuttle dysfunctional tau to [43]. The increase in expression levels of the autophagy-related protein Atg5 and the raised ratio between LC3A and LC3B in the total protein extracts in NIR-treated 3xTgAD mice suggests an increased induction of autophagy as reflected by increased production of autophagosomes. Atg5 is associated with the elongation of the autophagosomal membrane and the LC3B protein is necessary for the formation and closure of the autophagosome [25]. The increased expression of these proteins thus demonstrates the increased availability of important machinery involved in the degradation of dysfunctional tau in NIR light-treated animals, providing the opportunity for the increased clearance of the toxic protein. Together, these results provide insight into chaperone-mediated clearance and induced autophagy mechanisms that may be contributing to NIR light-induced reduction of tau. Many future studies can be performed to further the understanding of the application of NIR light treatment for AD. The investigation of other characteristics of AD pathology including neuroinflammation can provide a greater insight into the beneficial effects of NIR light treatments. In addition, alternative strategies for administration of NIR light can also be investigated to optimize the use of the treatment in human patients.

In conclusion, this study provided valuable evidence of the beneficial effects of NIR light treatments on the reduction of tau pathology and related cognitive dysfunction. The novel demonstration of this reduction of toxic tau species in the hTau mice, combined with the decrease of synaptic Aβ pathology in the Aβ/tau co-expressing 3xTgAD mice, further support the effectiveness of NIR light as a non-invasive treatment to reduce AD-related neuropathology and encourages its future clinical development.

## Methods

### Animals

Male and female hTau mice were utilized to measure in vivo levels of total tau and tau oligomers at the synapses and in the total protein extracts of the cortex and hippocampus regions (*n*=6, per experimental group). Female 3xTgAD (hAPP, tau P301L, presenilin-1) mice were used to measure Aβ and tau levels at the synapses and total protein extracts (*n*=7, per experimental group). Further, 3xTgAD mice were used to determine the induction of autophagy after NIR light treatment. Male 3xTgAD were not included in this study due to the known extensive variation in pathology development in the 3xTgAD male mice. The transgenic mice were 12-month-old at the initiation of the NIR light treatment schedule. Biochemical analysis was completed at the conclusion of the month-long treatment when the mice were 13-month-old. C57BL/6 wild-type male and female mice were employed to determine the ex vivo synaptic binding of tau oligomers, as well as the electrophysiological properties of brain slices from NIR light-treated mice challenged with ex vivo administration of tau oligomers (*n*=5, per experimental group, two slices per animal per experimental condition).

The experimental protocols performed in this study were approved and performed in accordance with the Institutional Animal Care and Use Committee of the University of Texas Medical Branch. All animals were housed under USDA standards (12:12 h light dark cycle, food and water ad libitum) at the UTMB vivarium.

### NIR Light Treatments

The NIR light treatments were performed in the same manner as previously described [19]. Briefly, a 90-s preprogramed, 670 nm wavelength light-emitting diode (LED) device, WARP10 (Quantum Devices, Barneveld, WI, USA), was held approximately 1 cm over from the top of the head while the body of the animal was covered with aluminum foil to localize treatment to the head. The control sham treatment group was held in the same manner with the LED device remaining off. The transgenic mice received one dose per day, 5 days a week for four consecutive weeks [15]. The wild-type mice received a condensed treatment schedule of 4 treatments per day over 5 days (20 total treatments in one week). As we previously described, the condensed treatment in wild-type mice resulted in similar alterations in binding properties as the 4-week treatment schedule [19]. Mice were utilized (behavior or sacrifice for tissue collection or brain slice preparation) immediately after the final NIR light treatment. At sacrifice, brains were quickly removed and used to prepare brain slices. The hippocampus and frontal cortex were collected and stored at −80 °C until further analyses were performed.

### Synaptosome Isolation

To isolate synaptosomes, the collected regional tissue was separately homogenized in Syn-PER synaptic protein extraction reagent (ThermoFisher) and underwent serial centrifugations, as per manufacturer’s instructions. Briefly, the homogenate was centrifuged at 1200x*g* for 10 min at 4 °C. A small fraction of the supernatant, consisting of the total protein extract, was collected for further biochemical analysis. The supernatant was transferred to a new tube and further centrifuged at 15,000x*g* for 20 min at 4 °C. The pellet formed after the second centrifugation contains the synaptosomal fraction. The pellet was resuspended in either HEPES-buffered Krebs-like (HBK) buffer, for binding experiments or radioimmunoprecipitation assay (RIPA) buffer, for Western blot and ELISA analysis. The synaptosome fractions are routinely analyzed by Western blot and electron microscopy to guarantee the quality of the preparation, as we have previously reported [44].

### ELISA Analysis of Total Tau

Total tau levels were measured by ELISA analysis using the total tau antibody, tau5 (ThermoFisher). For the ELISA, samples were incubated at 4 °C overnight on an ELISA plate with the coating buffer 0.1 M sodium bicarbonate (pH 9.6). The plates were then washed with Tris-buffered saline with low Tween 20 (0.01%) (TBS-low T) followed by blocking with 10% nonfat milk. The plates received another washing step followed by an incubation with tau5 antibody (1:1000 in 5% nonfat milk in TBS-low T; ThermoFisher) for 1 h at room temperature. Following a washing step, horseradish peroxidase-conjugated anti-rabbit IgG (1:10,000 in 5% nonfat milk in TBS-low T; Promega) was added to the plate and incubated for 1 h at room temperature. The plates were again washed with TBS-low T and 3,3,5,5-tetramethylbenzidine (TMB-1 component substrate; Sigma-Aldrich) was added. After 15 min, 1 M HCl was added to stop the reaction and the plate was read at 450 nm.

### Fluorescence Immunohistochemistry of Total Tau and Oligomeric Tau

Immunofluorescence was performed on post fixed (4% paraformaldehyde in 0.01 M PBS, pH 7.4) cryosectioned brain slices of the 3xTgAD and hTau mice that received NIR light or sham treatment. First, the slices were washed in phosphate-buffered saline (PBS) followed by permeabilized with 5% normal goat serum, 0.3% Triton X-100, and 0.05% Tween-20 in PBS for 1 h at room temperature. After a wash with PBS, the slides were incubated overnight at 4 °C with primary antibodies. The primary antibodies used were the tau oligomer specific antibody, anti-T22 (1:500; produced by Dr. Rakez Kayed [10]) and the total tau antibody, anti-tau 5 (1:1000, ThermoFisher). The slices were then washed with PBS and incubated with Alexa-conjugated secondary antibodies (1:400; Life Technologies) for 1 h at room temperature. Finally, the slices were washed in PBS and coverslips were mounted using Vectashield mounting medium containing DAPI (Vector Laboratories).

### Western Blot Analysis

Western blot analysis was performed on the total protein extracts and synaptosome fractions. Separation of the proteins in the samples obtained was done by 12% gradient SDS-polyacrylamide gel (HSP70 proteins) or 4–20% gradient gel (tau5, LC3A&B) electrophoresis. The separated proteins were transferred to a nitrocellulose membrane (Bio-Rad) and incubated with the specific antibody such as Tau5 (total tau; ThermoFisher), HSP70/HSP72, and HSC70 (Enzo Life Sciences) or LC3A&B (Cell Signaling) antibody overnight. The nitrocellulose membrane was then incubated with the appropriate fluorescent secondary antibody and imaged with an Odyssey infrared imager. The band densities were analyzed using Image J software, normalizing using the densities of the loading control obtained by reprobing the membranes for β-tubulin.

### RT-qPCR

Total RNA was isolated from the hippocampus of the sham- (*n*=7) and NIR light-treated (*n*=9) 3xTgAD mice utilizing the RNA isolation kit (QIAGEN). Real-time quantitative polymerase chain reaction (RT-qPCR) was conducted to determine mRNA levels of Atg5, as previously described [45]. The fold expression was calculated relative to the beta-actin gene.

### Tau Oligomer Preparation

Prepared recombinant tau oligomers were obtained by Dr. Rakez Kayed’s laboratory. The tau oligomers were produced as previously described [46]. Briefly, recombinant tau monomer protein was added to 1xPBS to obtain a concentration of 0.3 mg/ml. Aβ42 oligomers seeds were added to the tau mixture and incubated on an orbital shaker for 1 h at room temperature. The produced tau oligomers were used as seeds in a second batch of tau monomers to produce a new batch of tau oligomers. This protocol was repeated three times to ensure the elimination of the original Aβ seeds resulting in the production of tau oligomers. Each batch of oligomers is tested using dot blot with T22, a tau oligomer-specific antibody, Western blot analysis, and atomic force microscopy (AFM) to verify the quality of the tau oligomer preparation.

### Ex Vivo Tau Oligomer Binding to Synaptosomes

Synaptosomes isolated from mice receiving NIR light or sham treatment were resuspended in HBK buffer. Using the bicinchoninic acid (BCA) assay, 50 mg of total protein in our synaptosomal preparations was determined and aliquoted from each individual animal. Then, 50 nM of tau oligomers was then added to each synaptosome preparation and allowed to incubate for 1 h at room temperature. The samples were then centrifuged and washed with HBK buffer three times to thoroughly remove any unbound tau oligomers. The total protein levels were again measured by BCA and equal amounts of protein was analyzed by tau5 ELISA analysis, as described above.

### Electrophysiology

Long-term potentiation (LTP) measurements were performed as described previously [19]. Briefly, NIR light- and sham-treated wild-type mice were deeply anesthetized with isoflurane and transcardially perfused with 25–30 mL of room temperature carbogenated (95% O_2_ and 5% CO_2_ gas mixture) NMDG-ACSF. Further, 350 μm transverse brain sections containing Schaffer Collateral (SC) synapses were generated using Compresstome VF-300 (Precisionary Instruments, Greenville, NC). An initial protective recovery was done in the cutting solution at 32–34 °C for <12 min, then transferred to carbogen bubbling HEPES-ACSF recovery solution at room temperature. After recovery, slices were perfused in carbogen bubbling room temperature nACSF at a rate of approximately 3 mL/min. Treatments with oligomers occurred in the recovery phase where the slices were isolated to a separate chamber and incubated with the desired concentration of the oligomers for one hour. After the treatment, the slices were briefly washed by placing them in drug-free, oligomer-free recovery ACSF for 5 min before placing them on the recording stage. Using a horizontal P-97 Flaming/Brown micropipette puller (Sutter Instruments, Novato, CA), borosilicate glass capillaries were used to pull electrodes and filled with nACSF to get a resistance of 1–2 MΩ. Evoked field excitatory post synaptic potentials (fEPSPs) in the CA1 by stimulating SC were measured using HFS (3X100 Hz, 20 s) as described in our previous studies [19], digitized with Digidata 1550B (Molecular Devices, Sunnyvale, CA) and collected using an Axon MultiClamp 700B differential amplifier (Molecular Devices) connected to a Windows 7 computer (Dell Instruments, Round Rock, TX) running Clampex 10.6 software (Molecular Devices). Current stimulation was delivered through a digital stimulus isolation amplifier (A.M.P.I, ISRAEL) and set to elicit a fEPSP approximately 30% of maximum for synaptic potentiation experiments using platinum-iridium tipped concentric bipolar electrodes (FHC Inc., Bowdoin, ME). A stable baseline was obtained by delivering single pulse stimulation at 20 s interstimulus intervals. All data are represented as percentage change from the initial average baseline fEPSP slope, which was defined as the average slope obtained for the 10 min prior to HFS.

### Novel Object Recognition

After the conclusion of NIR light treatment regimen, the hTau mice underwent cognitive testing using the novel object recognition (NOR) paradigm. NOR consisted of three phases—(1) a habituation phase, (2) a training phase, and (3) a testing phase [47]. The habituation phase (two sessions of 10 min separated by 24 h) acclimated the animals to the experimenter and the environment. This was followed by the training phase, where the habituated animals were exposed to two identical objects placed in two quadrants at specific locations. After the animals explored the objects for 10 min, they were placed back in their home cages. After a retention interval of 24 h, novel object memory was tested, where the mice were exposed to one familiar and a novel object (different color and shape but sharing a common size and volume). The time spent exploring each object was recorded using ANY-Maze (Stoelting. Inc.) where an area 2 cm^2^ surrounding the object was defined such that nose entries within 2 cm of the object were recorded as time exploring the object. The percent time exploring each object (familiar versus novel) was reported as an object discrimination ratio (ODR) calculated by the following formula: ODR=(time exploring specified object) / (time exploring novel object + time exploring familiar object).

### Statistical Analysis

Data were statistically analyzed using SPSS software. Student’s *t* test was used to determine statistical significance in the tau level experiments. The calculated object discrimination ratios of the NOR behavior test were analyzed by the one-sample *t* test to determine the statistical variation from chance (0.50). The one-way ANOVA with Dunn’s post hoc test was used to determine statistical significance between the calculated fEPSP percentage of each condition in the electrophysiology experiment.
